# Determining Targets for Antiretroviral Drug Concentrations: A Causal Framework Illustrated With Pediatric Efavirenz Data From the CHAPAS‐3 Trial

**DOI:** 10.1002/pds.70051

**Published:** 2024-12-03

**Authors:** Michael Schomaker, Paolo Denti, Andrzej Bienczak, David Burger, Iván Díaz, Diana M. Gibb, Ann Sarah Walker, Helen McIlleron

**Affiliations:** ^1^ Department of Statistics Ludwig‐Maximilians Universität München München Germany; ^2^ Centre for Infectious Disease Epidemiology and Research, School of Public Health, University of Cape Town Cape Town South Africa; ^3^ Institute for Public Health, Medical Decision Making and HTA, UMIT TRIOL—University for Health Sciences and Technology Hall in Tirol Austria; ^4^ Division of Clinical Pharmacology, Department of Medicine University of Cape Town Cape Town South Africa; ^5^ Novartis Pharma AG Basel Switzerland; ^6^ Department of Pharmacy Radboud Insititute for Medical Innovation (RIMI), Radboud University Medical Center Nijmegen The Netherlands; ^7^ Division of Biostatistics, Department of Population Health New York University Grossman School of Medicine New York New York USA; ^8^ MRC Clinical Trials Unit at UCL, University College London London UK; ^9^ Wellcome Centre for Infectious Diseases Research in Africa (CIDRI‐Africa), Institute of Infectious Disease and Molecular Medicine, University of Cape Town Cape Town South Africa

**Keywords:** antiretroviral therapy, causal inference, continuous interventions, pediatrics, target concentrations

## Abstract

**Background:**

Determining a therapeutic window for maintaining antiretroviral drug concentrations within an appropriate range is required for identifying effective dosing regimens. The limits of this window are typically calculated using predictive models. We propose that target concentrations should instead be calculated based on counterfactual probabilities of relevant outcomes and describe a counterfactual framework for this.

**Methods:**

The proposed framework is applied in an analysis including longitudinal observational data from 125 HIV‐positive children treated with efavirenz‐based regimens within the CHAPAS‐3 trial, which enrolled children < 13 years in Zambia/Uganda. A directed acyclic graph was developed to visualize the mechanisms affecting antiretroviral concentrations. Causal concentration‐response curves, adjusted for measured time‐varying confounding of weight and adherence, are calculated using g‐computation.

**Results:**

The estimated curves show that higher concentrations during follow‐up, 12/24 h after dose, lead to lower probabilities of viral failure (> 100 c/mL) at 96 weeks of follow‐up. Estimated counterfactual failure probabilities under the current target range of 1–4 mg/L range from 24% to about 2%. The curves are almost identical for slow, intermediate and extensive metabolizers and show that a mid‐dose concentration level of ≥ 3.5 mg/L would be required to achieve a failure probability of < 5%.

**Conclusions:**

Our analyses demonstrate that a causal approach may lead to different minimum concentration limits than analyses that are based on purely predictive models. Moreover, the approach highlights that indirect causes of failure, such as patients' metabolizing status, may predict patients' failure risk, but do not alter the threshold at which antiviral activity of efavirenz is severely reduced.

**Summary**

- To determine therapeutic thresholds for antiretroviral drugs, it is common to develop predictive models to discriminate between patients with and without viral failure (or other outcomes), based on their individual characteristics. While these models are helpful in predicting high‐risk patients, they do not answer the more complex question of which concentration trajectories would most likely guarantee viral suppression.
- We propose that target concentrations should be calculated based on counterfactual probabilities of relevant outcomes, such as viral failure, and describe a counterfactual framework for this.
- This study illustrates the proposed framework in an analysis using pediatric data from the CHAPAS‐3 trial by calculating counterfactual failure probabilities for different plasma concentrations of efavirenz over time.
- Our results suggest that at the currently recommended lower threshold efavirenz concentration of 1 mg/L one has to likely expect relatively high failure probabilities (between 6% and 44%).
- The analyses demonstrate that a causal approach may lead to different (i.e., higher) minimum concentration limits than analyses that are based on purely predictive models.

## 1 Introduction

The overarching goal of combination antiretroviral therapy (cART) optimization is to choose the best combination of drugs, which are administered with doses and at time intervals providing the most optimal treatment outcomes. Recommended treatment plans can be disease specific (e.g., presence of co‐morbidities and co‐treatments), patient specific (e.g., age, weight, or pregnancy dependent), outcome specific (e.g., after treatment failure) or even individualized and drug specific (i.e., pharmacokinetic and pharmacodynamic properties of the drug could be taken into account because, for example, single nucleotide polymorphisms [SNPs] may alter a drug's metabolism or/and potency).

To propose an effective dosing regimen (possibly for different patient groups), it is essential to know the therapeutic window for maintaining drug concentrations within an effective and safe range [1]. For efavirenz, an antiretroviral drug used in both adults and children, it is typically suggested that mid‐dose concentrations (C_12h_) of ≥ 1 and ≤ 4 mg/L should be achieved as too low concentrations may be insufficient to guarantee viral suppression, and too high concentrations may lead to toxicities and negatively affect the central nervous system [2].

There is a rich literature on calculating the optimal efavirenz concentration thresholds that predict viral failure, both in adults and children [1, 3, 4, 5, 6, 7, 8]. However, the choice of these thresholds is controversial due to the different values derived from different studies, and also because many recommended thresholds for children were historically based on adult data [9]. Essentially all studies that suggest threshold values develop predictive models to discriminate between patients with and without viral failure, based on their individual concentrations and conditional on other individual characteristics such as the patient's metabolizing status, adherence patterns and demographics. In case of efavirenz, a particular emphasis in the model development is given to inclusion of SNPs in the CYP2B6 gene encoding the key metabolizing enzyme and leading to large between‐subject variability in systemic drug exposures.

While these models are certainly helpful in predicting high‐risk patients who require special attention, they do not answer the more complex question of which concentration trajectories would most likely guarantee viral suppression. It can be argued that the optimal dose regimen for efavirenz should be calculated such that it ensures drug concentrations for (almost) all patients well above the lowest threshold guaranteeing viral suppression (and not above an upper level which leads to strong side‐effects). To achieve this, a causal framework is needed because the underlying question is biological: specifically, what would the counterfactual probability of failure be at different concentrations (rather than what is the observed association between different concentrations and virological failure, as in models to date)—and which concentration would yield counterfactual failure probabilities that are low enough to be acceptable. Importantly, as highlighted in the causality literature, predictive models are defined on the observed data distribution, whereas counterfactual questions are answered based on post‐intervention distributions (i.e., failure distributions after hypothetically assigning doses/concentrations for *every* child) which requires additional context‐specific knowledge, in addition to statistical assumptions [10].

Here, we propose using a causal framework to estimate counterfactual virological failure probabilities for different possible efavirenz concentration trajectories. Concentration trajectories, which lead to too low probabilities of suppression, may then be used to define lower concentration thresholds which is the basis to calculate ideal doses with PK modelling. The suggested framework is applied to paediatric data from CHAPAS‐3, an open‐label, parallel‐group, randomised trial [11], where all data relevant to implement the approach is available, including plasma efavirenz concentrations, information on virological failure, adherence patterns, weight measurements and (socio‐)demographic data.

## 2 Methods

### 2.1 Study Setting

**FIGURE 1:**
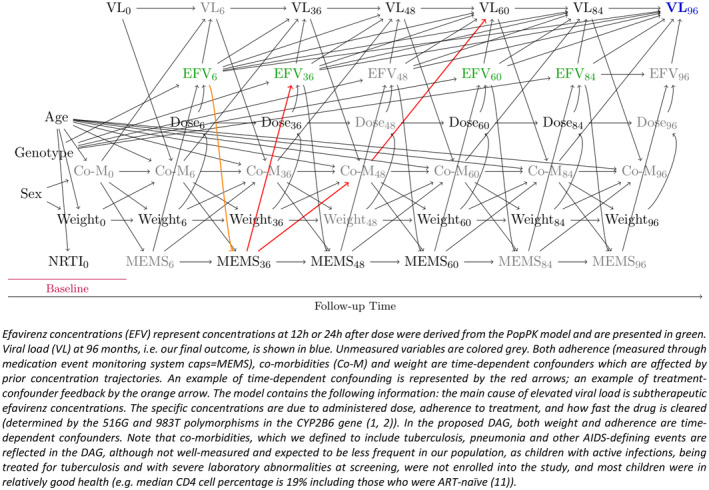
Directed acyclic graph, summarizing our knowledge of the clinical decision making and possible biological mechanisms.

As described previously, the Children with HIV in Africa–Pharmacokinetics and Adherence/Acceptability of Simple antiretroviral regimens study (CHAPAS‐3) enrolled 478 HIV‐infected children, under 13 years of age, in 4 sites in Uganda and Zambia [11]. Children in the study received cART comprising two nucleoside reverse transcriptase inhibitors (lamivudine and randomly assigned abacavir, or stavudine or zidovudine) and one non‐nucleoside reverse transcriptase inhibitor (efavirenz or nevirapine). We evaluated data of 125 children who received efavirenz [3]. Children were followed up at 6, 36, 48, 60, 84, and 96 weeks, with clinical assessments at every visit, viral loads (VL) at all timepoints except week 6, efavirenz levels 2–4 h after observed dose at all assessments other than week 48 and 96, and assessment of adherence through medication event monitoring system (MEMS) caps which record when medication bottles are opened (Figure 1). We used predicted efavirenz concentrations 12 and 24 h after dosing, calculated through a population PK (PopPK) model, in our analysis [1]. MEMS caps were initially planned to be used roughly between 0–18 and 54–72 weeks of follow‐up, though both funding constraints and practical considerations led to deviations. We neither imputed missing data, nor did we carry observations backwards to the last assessment or forwards to the next assessment. Viral failure was defined as > 100 copies/mL. In the trial, efavirenz dose was recommended to be based on weight using 200 mg for those weighing 10–13.9 kg, 300 mg for 14–19.9 kg, 400 mg for 20–34.9 kg, and 600 mg (the adult dose) above 34.9 kg, targeting a minimum around 14 mg/kg.

### 2.2 Target Concentrations

#### 2.2.1 Concept

In general, target concentrations for (antiretroviral) drugs are meant to quantify the levels at which a drug is both effective and safe. For efavirenz, one wants to determine which plasma concentrations are high enough such that antiviral activity prevents uncontrolled viral replication, and thus viral failure (and eventually a treatment switch). The minimum value which guarantees this for most patients is the lower target concentration limit (LTCL). Conversely, too high efavirenz concentrations may negatively affect central nervous system (CNS) outcomes. The upper target concentration limit (UTCL) defines where CNS outcomes are still acceptably low. In the trial, CNS events were, however, rare (18 events reported in 11 children): this is why we focus on the LTCL in the analyses below.

#### 2.2.2 Implications

The basis for deriving target concentrations, as defined above, has to be a causal estimand. This is because quantifying the effect of different concentration levels on viral outcomes is a strictly causal question that translates into asking: “what is the counterfactual probability of VL > 100 copies/mL at 96 weeks if *all* children had concentrations (12/24h after dose) of x mg/L at 6, 36, 60, and 84 weeks”, where x ranges from 0 to 10 mg/L". This question is different from asking: “What is the probability of failure at 96 weeks, among those with EFV concentrations of x mg/L at previous trial visits, and given a particular demographic and clinical profile before 96 weeks.” The former question is a causal question, the latter however is not, see Box 1 for more details.

BOX 1 **Predictive versus causal approach.**

**Table:** 

	Predictive approach	Causal approach
Estimand	Some function of *observed* data, e.g. “Probability of failure at 96 weeks, *among* those with EFV concentrations of x mg/L at previous trial visits, and *given* a particular demographic and clinical profile before 96 weeks.”^a^	Some function of *counterfactual* data, e.g. “Probability of failure at 96 weeks, if (possibly contrary to the fact) *every* patient had achieved to have a concentration of x mg/L throughout follow‐up.”^a^
Scientific question that can be answered	Rung 1^b^: A patient presents with a particular demographic and clinical profile, including measurements of EFV concentrations, at the follow‐up visit at 96 weeks. What is the predicted probability of failure given this specific profile?	Rung 2^b^: If a specific concentration trajectory (x_6_, …, x_96_) could be achieved, to which failure probability would the induced antiviral activity of efavirenz lead to in the given population? Would this probability differ by metabolizing status?
Statistical model	Required	Required
Causal model (DAG)	Not required	Required
Variable inclusion	Likely data‐adaptive	Structural: based on causal model and identification results
Statistical estimation	Any estimation technique that achieves a good‐variance bias tradeoff, i.e. reduces the prediction error Regression models are often useful	Any estimation technique that consistently estimates a probabilistic expression which is based on identification results that were derived using the assumptions encoded in the given the causal model G‐methods can address treatment‐confounder feedback and are a common choice [12]
Threshold choice (lower limit)	For example: dichotomize concentrations and pick the dichotomization cutoff that optimizes some (model‐selection) criterion *Rationale:* discriminate between patients with and without viral failure well, based on their individual concentrations and conditional on other individual characteristics—and reduce prediction error [1, 3, 7, 9]	Lowest concentration at which counterfactual failure probability is below *x*% *Rationale:* Determine at which level efavirenz “stops working” in the sense of reduced antiviral activity leading to viral failures
Aim	Predicting high‐risk patients who require special attention (using, among others, their concentration profile)	Understanding which concentration levels can lead to suboptimal treatment outcomes. In principle, population pharmacokinetic models can then be used to optimize dosing based on this knowledge

^a^ Formal definitions are given in Appendix S1.

^b^ According to Pearl's ladder of causation [10].

#### 2.2.3 Proposal

We propose the lowest concentration which guarantees that the counterfactual outcome probability is below *x*%, as the recommended lower target concentration limit. This differs from previous suggestions of dichotomizing concentrations and using these thresholds in a predictive (regression) model that optimizes some (model‐selection) criterion as LTCL [1, 3, 7, 9]. As described in Box 1 and Appendix S1, our proposal targets the actual scientific question implied by the concept of target concentrations and requires the application of causal inference concepts. This includes the development of a causal model, a structural approach to identify a valid adjustment set that can be used in the analysis, and appropriate statistical techniques [13].

### 2.3 The Causal Model

Figure 1 visualizes our proposed causal model, represented in a directed acyclic graph (DAG) which is explained in both the figure caption and in Appendix S2.

### 2.4 Identification

The assumptions required to use the observed study data to answer the causal question of interest are sequential conditional exchangeability, positivity and consistency [13, 14, 15]. The first assumption essentially requires no unmeasured confounders between viral failure and efavirenz concentrations at each time point. Applying the (generalized) back‐door criterion [16] to our causal model suggests that measuring weight and adherence is sufficient, both of which are at least partly available. Consistency relates to having a well‐defined intervention, which we consider to be unambiguous as defined above, although in practice concentrations can only be controlled through the given dose and maybe sometimes adherence (but not genotype). Positivity is the requirement that each concentration level of interest has a positive probability of occurring, given that a patient has followed the trajectory of interest so far and conditional on the covariate history. This assumption will always be violated to some degree for continuous variables; see the discussion for more details.

### 2.5 Statistical Analysis

For estimation, g‐methods are required, and regression techniques are insufficient. This is because of treatment‐confounder feedback; that is, the possibility that prior concentrations affect the confounders [13]. For example, adherence could potentially be affected by prior concentrations, as too high concentration values can cause nightmares and other central nervous system side effects, or strong discomfort, which might affect adherence patterns (Figure 1). We use the parametric g‐formula [12, 17, 18] for estimating the counterfactual failure probabilities, reported together with 95% compatibility intervals (CI) [19, 20]. The algorithm is described in Appendix S4. The LTCL is then determined as the lowest concentration at which the counterfactual failure probability is below 5%. We chose 5% as the threshold, rather than a value close to 0%, as VL > 100 copies/mL may occur due to subtherapeutic concentrations of partner NRTI drugs or replication in sanctuary sites with lower drug penetration than blood.

As adherence was infrequently measured, we used a single binary summary variable which indicated whether there was any sign of non‐adherence, defined as the mean memory caps opening percentage being smaller than 90%. Some data are likely missing for unmeasured reasons, for example due to technical issues will pill containers or missed visits related to caregiver's work responsibilities and lifestyle; thus, multiple imputation (MI) is invalid. In a separate theoretical paper [21], we show that under the missingness reasons reported by the pediatricians, a complete case analysis (CC) would nevertheless be valid for the given question based on the assumed DAG. Briefly, this is essentially because the reasons for missingness are not captured by variables which are relevant for identification, which yields a so‐called “closed missingness mechanism”, for which CC is preferred over MI [21].

To compare the proposed causal approach with a predictive approach, we repeated the analysis of Bienczak et al. [3]; that is, we fitted Cox proportional hazards model (recurrent event setup) for efavirenz dichotomization cutoffs from 0.2–4 mg/L, calculated Akaike information criteria (AIC) for each of those models and determined the LTCL as the cutoff which yielded the lowest AIC.

## 3 Results

Table 1 summarizes the data stratified by the number of VL > 100 c/mL between 36 and 96 weeks of follow‐up.

**TABLE 1:** Patient characteristics, stratified by number of observed VL > 100 c/mL occurring at or after week 36.

	All VL < =100 c/mL (*N* = 80)	One VL > 100 c/mL (*N* = 26)	Two or more VL > 100 c/mL (*N* = 19)	Overall (*N* = 125)
Sex				
Female	47 (59%)	11 (42%)	6 (32%)	64 (51%)
Male	33 (41%)	15 (58%)	13 (68%)	61 (49%)
Metabolizer group^a^				
Extensive metabolizers	28 (35%)	9 (35%)	3 (16%)	40 (32%)
Intermediate metabolizers	29 (36%)	10 (38%)	12 (63%)	51 (41%)
Slow metabolizers	23 (29%)	7 (27%)	3 (16%)	33 (26%)
Ultraslow metabolizers	0 (0%)	0 (0%)	1 (5%)	1 (1%)
Age (years, baseline)				
Median [Min, Max]	4.5 [2.0, 13.6]	3.91 [1.7, 11.3]	3.7 [2.6, 11.6]	4.29 [1.7, 13.6]
Randomized NRTI				
Stavudine	25 (31%)	11 (42%)	6 (32%)	42 (34%)
Zidovudine	29 (36%)	6 (23%)	5 (26%)	40 (32%)
Abacavir	26 (33%)	9 (35%)	8 (42%)	43 (34%)
Dose (baseline)				
200	26 (33%)	14 (54%)	8 (42%)	48 (38%)
300	39 (49%)	9 (35%)	9 (47%)	57 (46%)
400	14 (17%)	3 (11%)	2 (11%)	19 (15%)
600	1 (1%)	0 (0%)	0 (0%)	1 (1%)
BMQ score^b^				
Mean (SD)	8.7 (2.4)	8.7 (2.6)	7.9 (2.4)	8.6 (2.5)
Median [Min, Max]	9.3 [0.9, 14.6]	9.3 [0.4, 12.8]	8.5 [1.2, 10.7]	9.1 [0.4, 14.6]
SES summary^c^				
Mean (SD)	7.6 (2.3)	7.5 (2.4)	7.0 (2.0)	7.5 (2.3)
Median [Min, Max]	8.0 [3.0, 12.0]	7.0 [2.0, 11.0]	7.0 [3.0, 11.0]	7.0 [2.0, 12.0]
Missing	6 (8%)	1 (4%)	1 (5%)	8 (6%)
Weight (baseline)				
Median [Min, Max]	15.0 [10.0, 26.6]	13.9 [6.20, 29.0]	13.5 [9.60, 21.7]	14.5 [6.20, 29.0]
ART naive (baseline)				
Experienced	13 (16.3%)	1 (3.8%)	0 (0%)	14 (11.2%)
Naive	67 (83.8%)	25 (96.2%)	19 (100%)	111 (88.8%)
Available VL measurements				
Mean (SD)	4.3 (1.0)	4.1 (1.1)	4.5 (0.9)	4.2 (1.0)
Median [Min, Max]	4.5 [0, 5]	4.0 [1, 5]	5.0 [2, 5]	5.0 [0, 5]
Possible non‐adherence^d^				
Mean (SD)	8% (0.3)	4% (0.2)	11% (0.3)	7% (0.3)
Mean C12h across time points				
Median [Min, Max]	2.5 [0.7, 21.8]	2.2 [0.4, 20.2]	1.96 [0.6, 18.7]	2.3 [0.4, 21.8]
Missing	1 (1%)	0 (0%)	1 (5%)	2 (2%)
Mean C24h across time points				
Median [Min, Max]	1.7 [0.4, 20.4]	1.7 [0.2, 18.7]	1.3 [0.4, 17.8]	1.6 [0.2, 20.4]
Missing	1 (1%)	0 (0%)	1 (5%)	2 (2%)

^a^ Extensive metabolizers: 516 GG|983 TT; intermediate metabolizers: 516 GG|983 TC or 516 GT|983 TT; slow metabolizers: 516 TT|983 TT or 516 GT|983 TC; ultraslow metabolizers: 516 GG|983 CC.

^b^ Necessity‐Concern‐Score from the validated beliefs in medicine questionnaire (BMQ) [22]. The higher the values, the stronger the belief in the necessity of medicine, in relation to possible concerns.

^c^ Socioeconomic summary (SES) score: higher values indicate higher socio‐economic level. Maximum possible value: 14. Calculated as the sum of the (country‐specific) income quantile, education level (between 1 and 4), availability of electricity, availability of a toilet and 2*number of people per room (rounded).

^d^ Adherence as measured through a Medication Event Monitoring System (MEMS). Adherence is generally defined as the proportion of days with drug intake based on MEMS cap container openings. Reported values refer to signs of non‐adherence defined as < 90% of days with container openings, given that adherence measurements are available.

Compared to children recording VL > 100 c/mL twice or more often, children who were always observed to have VL ≤ 100 c/mL had, overall, slightly higher socio‐economic status, caregivers with stronger beliefs in the necessity of medicine, higher measured concentrations and adherence levels (but were otherwise broadly similar).

**FIGURE 2:**
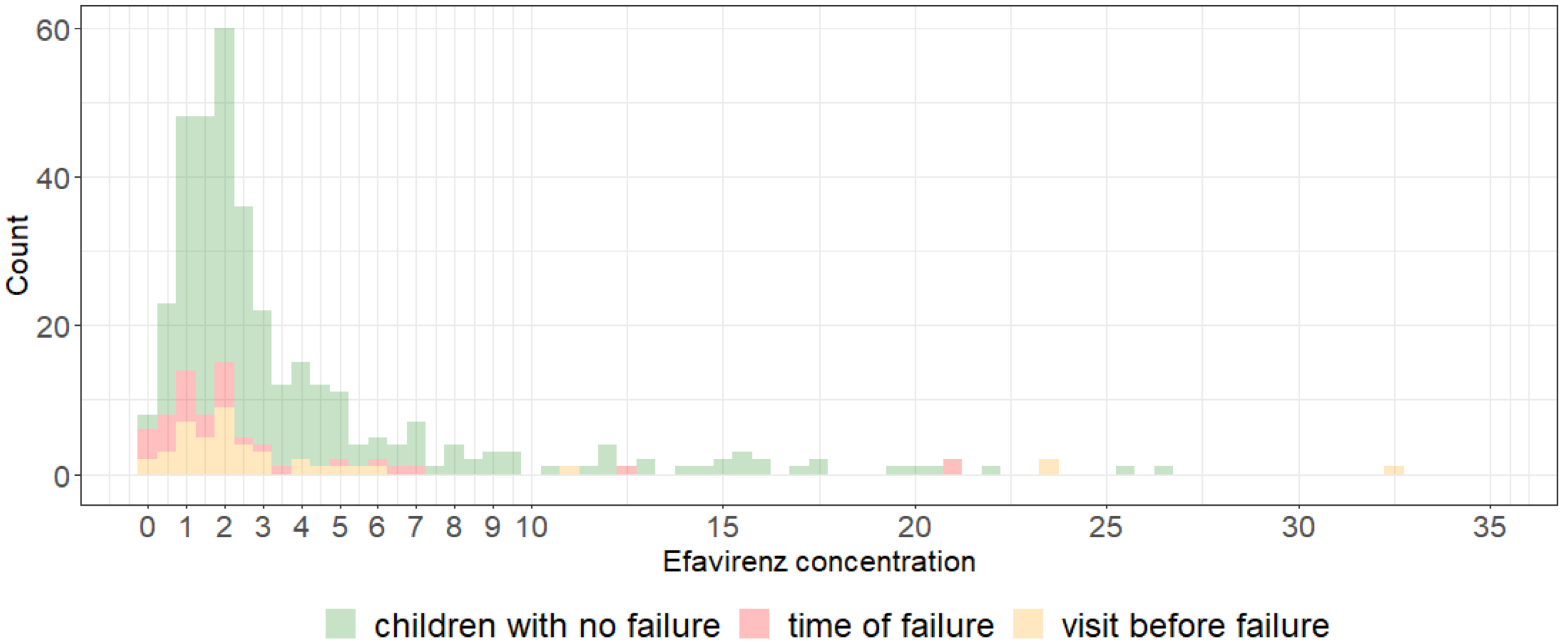
Efavirenz concentration values 12 h after dose (derived from PopPK model), at or before viral failure.

Thirteen children had at least one mid‐dose concentration measurement (i.e., 12 h after dose) recorded as higher than 15 mg/L, i.e., 11 mg/L above the current recommended upper target concentration. Of these 13 children, 11 were slow or ultraslow metabolizers. Four of these 11 patients were already receiving the lowest possible dose (200 mg tablet) at their first visit based on their weight. Figure 2 shows the distribution of all measured concentrations. At timepoints where children had VL > 100 c/mL (red color), few efavirenz concentrations were above 7 mg/L (only 6 measurements) and none were below 0.18 mg/L.

**FIGURE 3:**
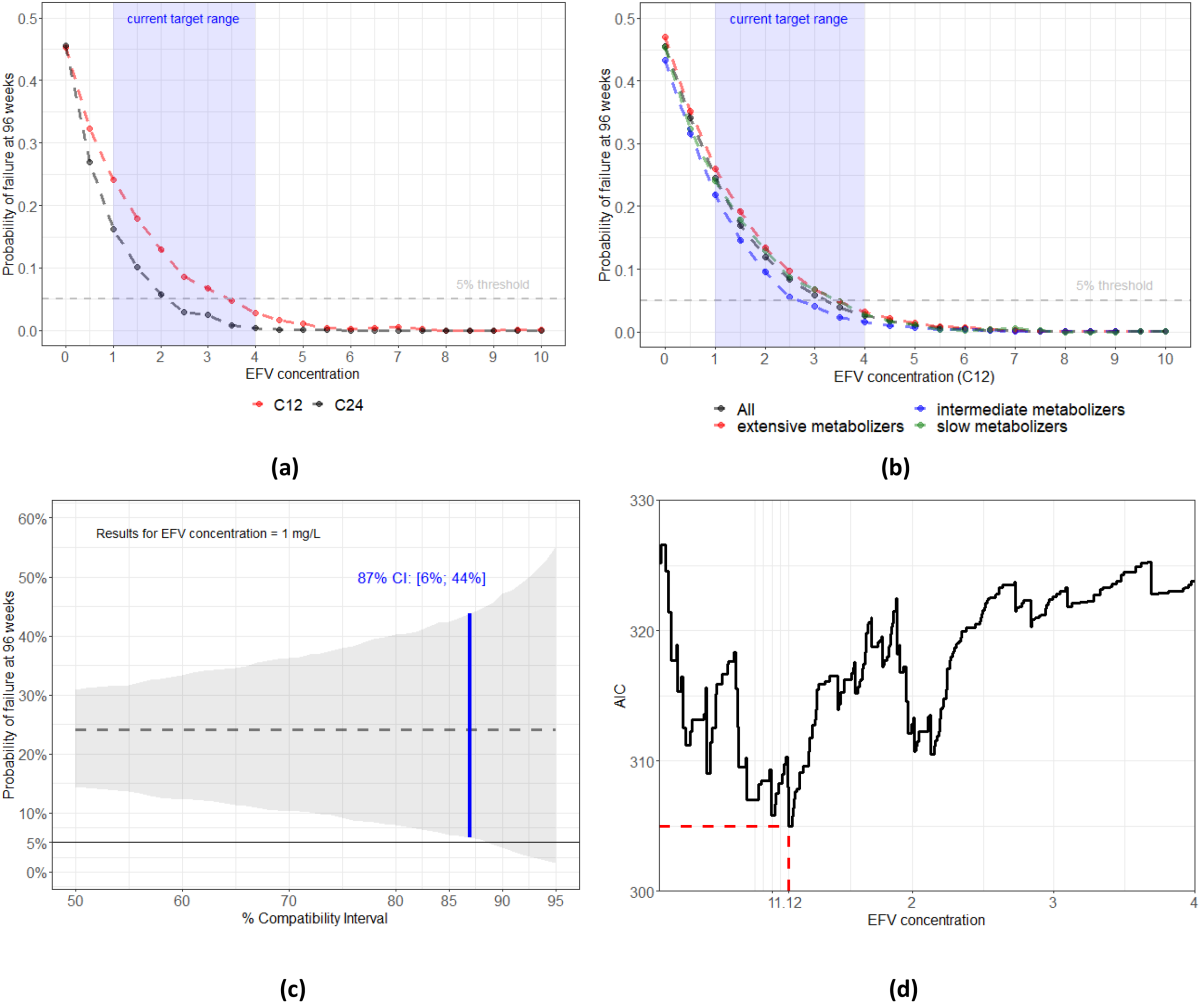
(a) Estimated causal concentration‐response curves (CCRC) when fixing efavirenz concentrations (12 and 24 h after dose) between 0 and 10 mg/L during the whole follow‐up and evaluating the counterfactual probability of failure at 96 weeks. (b) CCRCs, only 12 h after dose, stratified by metabolizing status. (c) 50%–95% compatibility intervals when C_12_ = 1 mg/L. The reported 87% compatibility interval highlights that at concentrations of 1 mg/L, viral failure (> 100 c/mL) probabilities between 6% and 44% are more compatible with the data than failure probabilities under 5%—given identification and estimation assumptions hold (d) AIC values for different efavirenz dichotomization cutoffs in Cox proportional hazards models. In the predictive approach the C_12_ LTCL is 1.12 mg/L, as this cutoff yields the lowest AIC.

The results of the counterfactual analyses are given in Figure 3. Higher efavirenz concentrations during follow‐up, 12 and 24 h after dose, lead to lower probabilities of VL > 100 c/mL (Figure 3a). The probabilities of VL > 100 c/mL rise sharply for low concentrations and suggest > 24% of viral failure probabilities at 96 weeks for a continuous exposure to mid‐dose (C_12_) concentrations at or below 1 mg/L. The calculated counterfactual failure probabilities in the current target range of 1–4 mg/L mid‐dose concentration range from 24% (95% CI: 2%; 55%) to about 2% (95% CI: 0%, 13%). Naturally, 24 h concentrations are lower than 12 h concentrations—and therefore lead to different probabilities of VL > 100 c/mL for the same concentration. To achieve a probability of VL > 100 c/mL 5% or lower (dashed line), a mid‐dose concentration of about 3.5 mg/L, or higher, would be required (corresponding to a C_24_ concentration level > 2.5 mg/L). This is the LTCL derived by the causal approach, which is higher than the C_12_ LTCL limit of 1.12 mg/L obtained by the predictive approach (Figure 3d).

Results did not differ with respect to slow, intermediate and extensive metabolizers (Figure 3b). Owing to the relatively small sample size, the 95% compatibility intervals were wide (Figure 3c, Appendix S1). However, under the identification and estimation assumptions stated above, for the currently recommended lower target concentration limit of 1 mg/L failure probabilities between 6% and 44% are more compatible with the data than other values, such as low failure probabilities < 5%: Figure 3c shows which failure probabilities at 1 mg/L are more compatible with our data, by varying the width of the CIs. Note that the figure corresponds to a *p*‐value function [20], with different varying target hypotheses on the *y*‐axis and CI levels instead of *p*‐values on the *x*‐axis.

The “natural course scenario for our g‐formula analyses did not exhibit any signs of strong model mis‐specification or unmeasured confounding” (Figure S2).

## 4 Discussion

### 4.1 Statement of Principal Findings

We have argued that antiretroviral target concentrations, which can be used for dose optimization, should be based on counterfactual probabilities of virological suppression. Our analyses, using a causal framework, show that such an approach may yield different (i.e., higher) lower target concentration limits than analyses that are based on purely predictive models.

### 4.2 Interpretation of Results

Unsurprisingly, our results indicate lower failure probabilities with higher efavirenz concentrations. As we have calculated our results in absolute rather than in relative terms, one can see that optimized treatment plans can likely reduce the probability of failure close to 0%. Moreover, the steep incline in failure risk for low concentrations below 2 mg/L indicates that the antiviral activity of efavirenz is severely reduced at a certain threshold.

An important finding relates to the essentially identical concentration‐response curves for slow, intermediate and extensive metabolizers. This can be explained with the developed DAG: the metabolizer status determines (predicts) who has fast clearance of efavirenz and thus low concentrations and higher risk of failure; but if we could “control” the concentration at a particular level, then the metabolizer status does not change anything anymore, as it does not have a direct effect on failure, just an indirect one through the concentration. This highlights again the crucial distinction between predictive and causal viewpoints. Practically however, it raises an important consideration: given (i) the lack of availability of point‐of‐care (POC) tests to determine metabolizer status and (ii) the fact that we can “control” concentrations predominantly through dosing guidelines, should recommended doses target a standardized “average” across genotypes (hence risking virological failure in fast metabolizers) or target the “minimum” across genotypes (hence risking toxicity/poor adherence in slow metabolizers)?

### 4.3 Results in Context

The study originally suggesting a target range of 1–4 mg/L, reported on early data in 2001, was pragmatic in their associative approach and actually focused on the upper threshold of 4 mg/L to avoid central nervous system side effects [8]. Both the study from 2001, and others [5, 6, 7, 9], were based on adult data and reported higher C_12_ and C_24_ concentrations than our paediatric study. In contrast to our data (Figure 2), it has been noted in studies such as the ENCORE1 trial, that not many adult patients with virological failure had efavirenz concentrations < 1 mg/L. Other publications using the same pediatric data as us noted these differences between children and adults and determined ideal thresholds (e.g., 1.12 mg/L for C12h) based on model selection criteria, including the Akaike Information Criterion [3].

While *we used the same data as Bienczak* et al. [3], our analytical approach suggests somewhat higher values for the lower limit of the target concentration range (i.e., higher than 1.12 mg/L for C12h). Although our results were imprecise, the reported compatibility intervals highlight that at concentrations of 1 mg/L, viral failure (> 100 c/mL) probabilities between 6% and 44% are more compatible with the data than failure probabilities under 5%—given our identification and estimation assumptions hold (see limitation section below). It follows that, despite the imprecision and limitations—and the illustrative nature of our analyses— one may be cautious in adopting a lower threshold at, or under, 1 mg/L. As explained in Box 1, the differences in the derived thresholds can be attributed to the fact that we used a causal, rather than a predictive approach.

The consequences of using a counterfactual target concentration approach would be seen when developing dosing guidelines. For example, Bienczak et al. [1] suggested modified dosing strategies for children, stratified by metabolizer status and weight‐bands. Their proposal highlights the fact that for (ultra)slow metabolizers doses as low as 50 mg can be sufficient for light children. Simulations with their PK models showed that a low proportion of children would achieve concentration below 1 mg/L with the suggested dosing strategy—but if a different lower threshold would be assumed, this may affect the final strategy and thus recommendations.

Optimal thresholds using predictive models have been developed for other antiretrovirals too. Moholisa et al., for example, calculated thresholds for children using nevirapine and lopinavir [23, 24]. Our suggested strategy could be readily employed to derive target concentrations for these drugs and for other settings too. It would then however be important to adapt the directed acyclic graph, to reflect the respective differences with respect to the role of metabolic components, existing dosing decisions, and outcomes. It may be noted that typical time‐dependent confounders, which would ideally be measured, are weight and adherence and the basic structure of our DAG may be appropriate.

### 4.4 Strengths and Limitations

A particular strength of our analyses is the availability of a relatively large amount of variables from a controlled trial where standardized assessments, including of weight, dose, and adherence, were conducted at regular pre‐specified intervals—in contrast to observational studies with far less standardization—and parallel pharmacokinetic studies could be conducted. Moreover, we have been using state‐of‐the‐art causal inference estimation methods, in conjunction with a causal graph, to be able to target a counterfactual question.

There are two major limitations: first, despite being larger than intensive pharmacokinetic studies, the sample was still relatively small and there was some missing data, both of which made our estimates imprecise. It would be important to extend our analyses to a bigger sample, maybe by pooling data over different studies with modern data fusion techniques [25]. While the results of the current analysis suggest that increasing the minimum efficacy threshold for efavirenz might be required to ensure favorable treatment outcomes, our results should be validated using a dataset comprising a larger patient population. It is also important to note that missing data in adherence measurements occurred due to missed visits, breaking of the memory cap containers and because the design of the trial meant that each caregiver had them for specific intervals of time. We therefore had to use an adherence summary measure, which means that some unmeasured confounding may still be present and bias due to missing data in general cannot be excluded, despite careful consideration of this aspect. Additionally, it cannot be ruled out that there is unmeasured confounding due to other reasons (e.g., malnourishment).

Another relevant limitation relates to positivity violations for very small and high concentration levels: to trust estimates below 0.5 mg/L, one has to hope that extrapolation of our models, used in the g‐formula, worked well. Alternatively, to avoid the positivity assumption, one could change the question of interest and fix target concentrations to specific levels only for patients where this seems likely given patient's individual covariates, as we described recently [15].

### 4.5 Conclusions

Our analysis is a compelling example of how a causal framework can be applied to derive a target concentration threshold for efficacy of antiretroviral drugs. We demonstrated that this approach could result in potentially different estimates to the ones derived through purely predictive approaches (e.g., the 1.12 mg/L derived by Bienczak et al. on the *same* data [3])—though imprecision, missing data and remaining unmeasured confounding due to non‐adherence suggest some caution regarding our estimates. We envisage that employing causal graphs and g‐methods will play an increasing role in defining the concentration‐response curves across multiple therapeutic areas in the future.

### 4.6 Plain Language Summary

Different people who take the same drug dose, may still have different drug concentrations in their blood, for example because of their individual metabolism. Patients exposed to suboptimal concentrations may experience negative outcomes. Predicting the likelihood of negative outcomes for different patient groups is a different question to predicting at which concentration the drug stops working well. The latter is a cause‐effect question about which concentration leads to a negative outcome, on average, when looking at the whole population. Currently, recommended concentration intervals for antiretroviral drugs, which are used to treat people who acquired HIV, are based on predictive considerations and not a cause‐effect relationship. We describe how to follow the latter approach. We illustrate this approach in an analysis of 125 HIV‐positive children treated with efavirenz as part of the CHAPAS‐3 trial, which enrolled children < 13 years in Zambia/Uganda. In this analysis, the probability of viral failure (a negative outcome) is relatively high (between 6% and 44%) at the currently recommended lower concentration threshold of 1 mg/L. Our findings suggest that using a method based on understanding cause and effect relationships could lead to different recommendations for concentration intervals compared to using predictive models alone.

## Author Contributions

The study was designed by M.S., P.D. and H.M. Data was collected and interpreted by A.B., D.B., D.M.G., A.S.W., P.D., H.M. All authors reviewed the study design and interpreted the data. Methods development was led by M.S. and I.D. The first draft of the article was written by M.S. All authors reviewed this and the following drafts, revising it critically for its content. All authors have read and approved the final version and agree with the manuscript's conclusion.

## Ethics Statement

The CHAPAS‐3 trial was approved by Research Ethics Committees in Zambia, Uganda, and the UK.

## Conflicts of Interest

The authors declare no conflicts of interest.

## Supporting information


Appendix S1.
