# Supplementary material for: Determining Targets for Antiretroviral Drug Concentrations: A Causal Framework Illustrated With Pediatric Efavirenz Data From the CHAPAS‐3 Trial
Source: Pharmacoepidemiol Drug Saf. 2024 Dec 3;33(12):e70051. doi: 10.1002/pds.70051 (PMC11614751; doi:10.1002/pds.70051)
Supplement: Supplementary file 1 — Appendix S1. [file PDS-33-e70051-s001.pdf]

Appendix S1: Technical Details

General notation: We consider a longitudinal data setup where at each trial visit t, $t = 0, 6, 36, 48, 60, 84, 96$, we are interested in an outcome Y_t, a continuous intervention A_t and covariates L_t^j, $j = 1, \ldots, q$ for $i = 1, \ldots, n$ individuals. We denote $\mathbf{L_0} = \{L_0^1, \ldots, L_0^{q_0}\}$ as "baseline variables" and $\mathbf{L}_t = \{L_t^1, \ldots, L_t^q\}$ as follow-up variables, with $q, q_0 \in \mathbb{N}$. The intervention and covariate histories of a unit i (up to and including time t) are $\bar{A}_{t,i} = (A_{0,i}, \ldots, A_{t,i})$ and $\bar{L}^s_{t,i} = (L^s_{0,i}, \ldots, L^s_{t,i})$, $s = 1, ..., q$, $i = 1, \ldots, n$, respectively. We are interested in the counterfactual outcome $Y_{t,i}^{\bar{a}_t}$ that would have been observed at time t if unit i had received, possibly contrary to the fact, the intervention history $\bar{A}_{t,i} = \bar{a}_t$, $a_{t,i} \in \mathbb{R}$.

Specific Data: Measured baseline variables include, among others, $\mathbf{L_0}$ = sex, metabolism status, age, NRTI regimen, weight, viral load. Measured follow-up variables are $\mathbf{L}_t$ = weight, adherence (measured through memory caps, MEMS), dose; though the latter does not need to be included in the adjustment set. The outcome Y_t is elevated viral load, and with A_t we refer to the efavirenz plasma concentration at time t.

Estimands: Following Box 1, shown in the manuscript, we can distinguish different estimands:

- A predictive estimand (rung 1) could be defined along the question: "What is the probability of failure at 96 weeks, among those with EFV concentrations of a mg/L at previous trial visits, and given a particular demographic and clinical profile before 96 weeks". Formally, this corresponds to

$$P(Y_{96} = 1 \mid \bar{A}_t = (a, \ldots, a), \bar{l}_t)$$

- The causal estimand (rung 2) considered in the paper is essentially the longitudinal concentration response curve, i.e.

$$\bar{a}_t \mapsto P(Y_{96}^{\bar{a}_{96}} = 1) \qquad \forall \bar{a}_{96} \in \bar{\mathcal{A}}_{96}, , \tag{1}$$

  with $\bar{\mathcal{A}}_{96} = \{(0, 0, \ldots, 0), \ldots, (10, 10, \ldots, 10)\}$. It answers the following question: "If a specific concentration trajectory $\bar{a}_{96}$ could be achieved, to which counterfactual failure probability $P(Y_{96}^{\bar{a}_{96}} = 1)$ would the induced antiviral activity of efavirenz lead to in the given population?"

- Causal estimands that are on rung 3 of Pearl's ladder of causation are not considered in the paper. An example for a rung 3 estimand would be one that is defined through the following question: "A group of patients experienced virological failure at 96 weeks and had efavirenz concentrations of 1 mg/L throughout follow-up. Would they not have failed after 96 weeks if they had had plasma concentrations of 4 mg/L ?".

Appendix S2: More details on the structural model

The DAG reflects the consideration that viral failure is essentially caused by not enough plasma efavirenz concentration in the body. Alternative reasons for failure may be subtherapeutic concentrations of other antiretroviral drugs (lamivudine, abacavir, stavudine, zidovudine) and there might be interactions with co-morbidites and co-medications. The latter is represented in the DAG through the arrow Co-M$_t \to$ VL$_{t+1}$; the former is not shown as the effect of other drug concentrations on viral failure is not of interest in our analysis.

Given the short half-life of the drug relative to the measurement interval, no arrow from EFV$_t$ to EFV$_{t+1}$ is required. Pediatricians were advised to assign doses based on weight bands, as recommended by WHO (Weight$_t \to$ Dose$_t$).

Additionally, it is important to note that co-morbidities, as reflected in the DAG, are less frequent in this data analysis due to trial inclusion criteria, which excluded children with active infections, those receiving tuberculosis treatment, and those with laboratory abnormalities. Both weight and MEMS (representing adherence) are considered time-dependent confounders influenced by prior treatments (i.e., drug concentrations). Weight indirectly impacts concentration through dosing, while adherence directly

affects it. Adherence itself is affected by prior concentrations, as too high concentration values can cause nightmares and other central nervous system side effects, or strong discomfort, that might affect adherence patterns. Weight is affected from prior concentration trajectories through the pathway of viral load and co-morbidites. Lastly, both weight and adherence affect viral outcomes not only through EFV concentrations, but potentially also through co-morbidities such as malnutrition, pneumonia and others.

Appendix S3: Additional analysis results

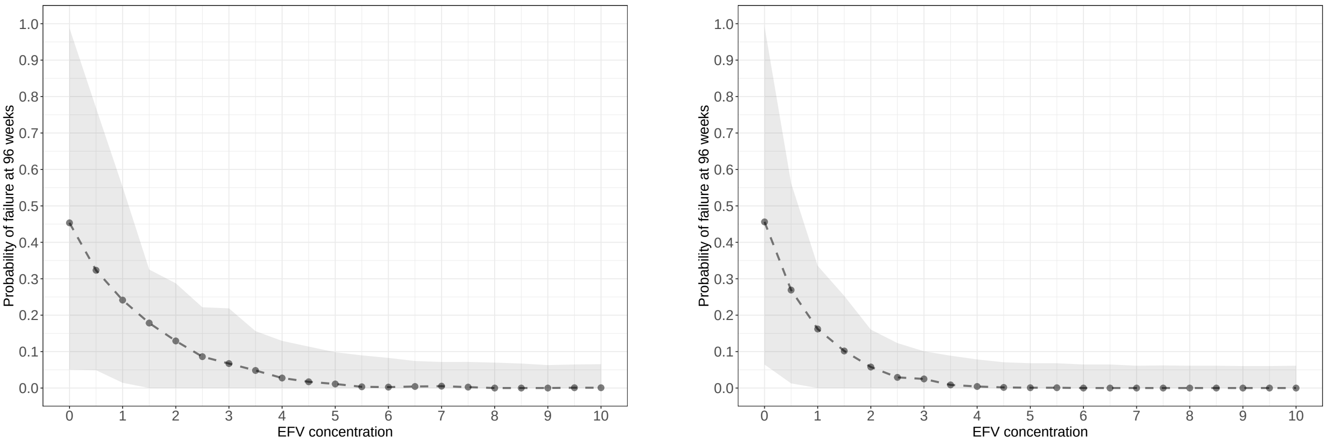

(a) (b)

Figure S1: Estimated Causal Concentration-Response Curves when fixing efavirenz concentrations (a) 12h after dose (b) 24h after dose between 0 and 10 mg/L during the whole follow-up and evaluating the counterfactual probability of failure at 96 weeks; presented with 95% bootstrap confidence intervals.

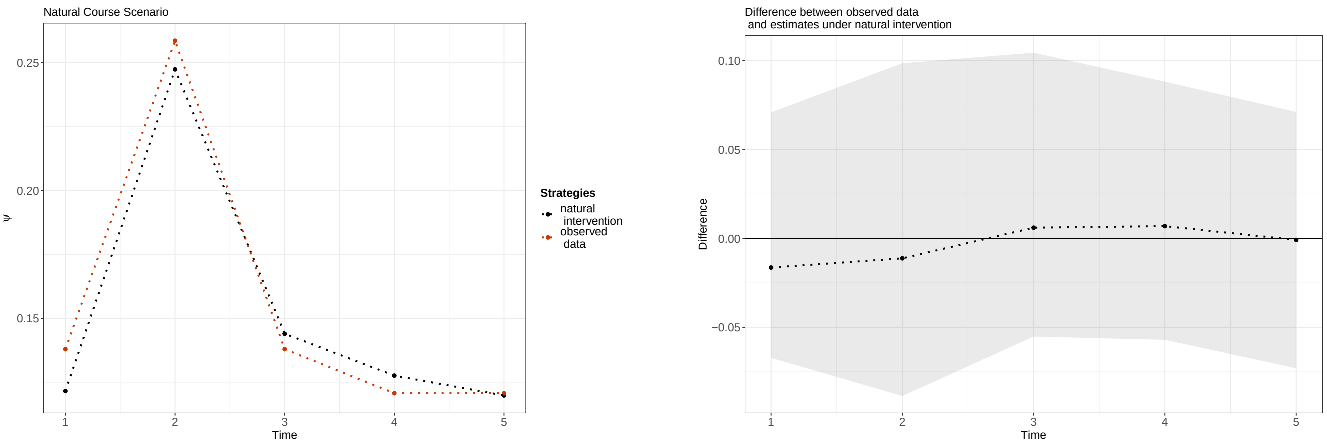

(a) (b)

Figure S2: Natural course scenario for the g-formula analysis, and for efavirenz concentrations 12h after dose. (a) The probability of failure is shown during follow-up, both for the observed data and the "natural intervention". Panel (b) shows the difference between the two curves (including 95% CI) from panel (a); this difference should be close to zero under the assumptions of no model mis-specification and no unmeasured confounding.

Appendix S4: Estimation algorithm based on the g-formula

In step 1 we model the outcome (viral load) and confounder (specifically, weight) models with linear and logistic additive regression models. This approach allows the inclusion of (penalized) splines and non-linear interactions and thus flexible extrapolation in "regions" with no, or low, support for concentration trajectories of interest. The respective covariate sets include initially all baseline variables and variables needed for identification (adherence, weight), which are measured prior to the respective outcome/confounder (we then use model selection to reduce the variable set, to achieve a good bias-variance tradeoff). Then, in step 2, a counterfactual dataset is created, where a) the concentration values are set to a fixed value at each time point (e.g., 3 mg/L) and b) the counterfactual outcomes, and confounders, are predicted based on both this data set and the models fitted in step 1. The counterfactual failure probabilities can then (step 3) simply be calculated as the mean of the simulated counterfactual outcomes. This procedure is then repeated (step 4) for concentration values of 0 to 10 mg/L, in steps of 0.5 mg/L, the estimates are visualized in a curve at 96 weeks of follow-up and bootstrapping (step 5) is used to calculate 95% compatibility intervals (CI). Those CI's are numerically identical to what have been traditionally called "confidence intervals" but offer refined interpretations: the CIs summarize how compatible different hypothesized failure probabilities are with the data, given our model assumptions are correct.
